# Correction: The association between the risk perceptions of COVID-19, trust in the government, political ideologies, and socio-demographic factors: A year-long cross-sectional study in South Korea

**DOI:** 10.1371/journal.pone.0314924

**Published:** 2024-12-02

**Authors:** Yo Han Lee, Hyun-Hee Heo, Hyerim Noh, Deok Hyun Jang, Young-Geun Choi, Won Mo Jang, Jin Yong Lee

There are errors in the Funding statement. The correct Funding statement is as follows: A grant (K2225791) from Korea University supported YHL’s work. H-HH’s work was supported, in part, by the Ministry of Education of the Republic of Korea and the National Research Foundation of Korea (NRF-2022S1A5B5A16057001). YGC’s work was supported, in part, by 2020R1G1A1A01006229 awarded by the National Research Foundation of Korea. However, any funder did not play any role in the study design, data collection, analysis, interpretation, and publication decision.

There is an error in affiliation 5 for author Young-Geun Choi. The correct affiliation 5 is: Department of Mathematics Education, Sungkyunkwan University, Seoul, Republic of Korea. Young-Geun Choi’s email address is: ygchoi@skku.edu
