# Supplementary material for: The association between the risk perceptions of COVID-19, trust in the government, political ideologies, and socio-demographic factors: A year-long cross-sectional study in South Korea
Source: PLoS One. 2023 Jun 21;18(6):e0280779. doi: 10.1371/journal.pone.0280779 (PMC10284396; doi:10.1371/journal.pone.0280779)
Supplement: S1 File — (DOCX) [file pone.0280779.s001.docx]

**Patterns and trends in factors associated with affective and cognitive risk perceptions of COVID-19**

**S1 Table. Details of the survey**

| Survey | Period | Respondents sampled, n | Respondents successfully  interviewed, n | Response rate (%) |
| --- | --- | --- | --- | --- |
| 1 | 02/04/20 – 02/06/20 | 1,000 | 6,233 | 16% |
| 2 | 02/11/20 – 02/13/20 | 1,001 | 7,052 | 14% |
| 3 | 02/18/20 – 02/20/20 | 1,002 | 7,673 | 13% |
| 4 | 02/25/20 – 02/27/20 | 1,001 | 6,614 | 15% |
| 5 | 03/03/20 – 03/05/20 | 1,000 | 6,853 | 15% |
| 6 | 03/10/20 – 03/12/20 | 1,001 | 6,549 | 15% |
| 7 | 03/17 /20– 03/19/20 | 1,000 | 7,231 | 14% |
| 8 | 03/24/20 – 03/26/20 | 1,001 | 7,392 | 14% |
| 9 | 03/31/20 – 04/02/20 | 1,002 | 7,304 | 14% |
| 10 | 04/21/20 – 04/23/20 | 1,001 | 7,954 | 13% |
| 11 | 05/06/20 – 05/07/20 | 1,004 | 7,147 | 14% |
| 12 | 05/19/20 – 05/21/20 | 1,000 | 8,007 | 13% |
| 13 | 06/02/20 – 06/04/20 | 1,001 | 7,716 | 13% |
| 14 | 06/16/20 – 06/18/20 | 1,001 | 8,536 | 12% |
| 15 | 06/30/20 – 07/02/20 | 1,000 | 8,976 | 10% |
| 16 | 07/14/20 – 07/16/20 | 1,001 | 7,041 | 14% |
| 17 | 08/18/20 – 08/20/20 | 1,002 | 6,280 | 16% |
| 18 | 09/15/20 – 09/17/20 | 1,000 | 6,201 | 16% |
| 19 | 10/20/20 – 10/22/20 | 1,001 | 6,173 | 16% |
| 20 | 11/17/20 – 11/19/20 | 1,001 | 6,029 | 17% |
| 21 | 12/08/20 – 12/10/20 | 1,000 | 6,273 | 16% |
| 22 | 01/18/21 – 01/21/21 | 1,000 | 6,597 | 15% |
| 23 | 02/16 /21– 02/18/21 | 1,000 | 6,841 | 15% |

**S2 Table. Details of each phase**

| Time point | Period (Survey) | The number of confirmed cases on survey week (each survey) | Meaning (event-based) |
| --- | --- | --- | --- |
| Phase 1 | 01/02/20 – 02/17/20 (1–2) | 10, 4 | Novel COVID-19 spread in Korea |
| Phase 2 | 02/18/20 – 05/05/20 (3–10) | 573, 3,134, 3,578, 848, 799, 622, 275, 77 | First community outbreak |
| Phase 3 | 05/06/20 – 08/11/20 (11–16) | 108, 141, 311, 317, 380, 292 | Transition to weakened social distancing |
| Phase 4 | 08/12/20 – 11/12/20 (17–19) | 2,150, 760, 680 | Gwanghwamun mass rally and social distancing in the metropolitan area upgraded |
| Phase 5 | 11/13/20 – 07/06/21  (20–23) | 2,235, 5,323, 2,792, 3,455 | COVID-19 spread again |

**S3 Table. General characteristics of respondents by phase**

|  |  | Overall |  | Phase 1 |  | Phase 2 |  | Phase 3 |  | Phase 4 |  | Phase 5 |  |
| --- | --- | --- | --- | --- | --- | --- | --- | --- | --- | --- | --- | --- | --- |
| N | (Respondents) | 23,018 |  | 2,001 |  | 8,006 |  | 6,007 |  | 3,003 |  | 4,001 |  |
| Age (years) | |  |  |  |  |  |  |  |  |  |  |  |  |
|  | Mean | 49.3 |  | 49.3 |  | 49.2 |  | 49.1 |  | 49.5 |  | 49.7 |  |
|  | SD | 16.7 |  | 16.4 |  | 16.5 |  | 17.0 |  | 16.7 |  | 17.0 |  |
|  | 19–29 | 3,630 | 15.8% | 302 | 15.1% | 1,221 | 15.3% | 993 | 16.5% | 460 | 15.3% | 654 | 16.3% |
|  | 30–39 | 3,505 | 15.2% | 324 | 16.2% | 1,251 | 15.6% | 904 | 15.0% | 457 | 15.2% | 569 | 14.2% |
|  | 40–49 | 4,397 | 19.1% | 382 | 19.1% | 1,587 | 19.8% | 1,123 | 18.7% | 567 | 18.9% | 738 | 18.4% |
|  | 50–59 | 4,802 | 20.9% | 423 | 21.1% | 1,661 | 20.7% | 1,253 | 20.9% | 627 | 20.9% | 838 | 20.9% |
|  | 60+ | 6,684 | 29.0% | 570 | 28.5% | 2,286 | 28.6% | 1,734 | 28.9% | 892 | 29.7% | 1,202 | 30.0% |
| Gender | |  |  |  |  |  |  |  |  |  |  |  |  |
|  | Men | 11,613 | 50.5% | 1,017 | 50.8% | 4,055 | 50.6% | 3,014 | 50.2% | 1,500 | 50.0% | 2,027 | 50.7% |
|  | Women | 11,405 | 49.5% | 984 | 49.2% | 3,951 | 49.4% | 2,993 | 49.8% | 1,503 | 50.0% | 1,974 | 49.3% |
| Job | |  |  |  |  |  |  |  |  |  |  |  |  |
|  | Unemployed | 2,722 | 11.8% | 219 | 10.9% | 883 | 11.0% | 735 | 12.2% | 375 | 12.5% | 510 | 12.7% |
|  | Farming/Forestry/Fishing | 682 | 3.0% | 59 | 2.9% | 221 | 2.8% | 184 | 3.1% | 97 | 3.2% | 121 | 3.0% |
|  | Self-employed | 3,339 | 14.5% | 259 | 12.9% | 1,219 | 15.2% | 844 | 14.1% | 426 | 14.2% | 591 | 14.8% |
|  | Blue-collar | 3,552 | 15.4% | 341 | 17.0% | 1,158 | 14.5% | 966 | 16.1% | 455 | 15.2% | 632 | 15.8% |
|  | White-collar | 7,051 | 30.6% | 626 | 31.3% | 2,542 | 31.8% | 1,782 | 29.7% | 943 | 31.4% | 1,158 | 28.9% |
|  | Homemaker and Student | 5,579 | 24.2% | 489 | 24.4% | 1,948 | 24.3% | 1,470 | 24.5% | 694 | 23.1% | 978 | 24.4% |
| Self-reported household status | |  |  |  |  |  |  |  |  |  |  |  |  |
|  | Upper/Upper Middle | 3,617 | 15.7% | 327 | 16.3% | 1,211 | 15.1% | 966 | 16.1% | 467 | 15.6% | 646 | 16.1% |
|  | Middle | 10,331 | 44.9% | 928 | 46.4% | 3,663 | 45.8% | 2,651 | 44.1% | 1,307 | 43.5% | 1,782 | 44.5% |
|  | Lower Middle/Lower | 9,070 | 39.4% | 746 | 37.3% | 3,132 | 39.1% | 2,390 | 39.8% | 1,229 | 40.9% | 1,573 | 39.3% |
| Residential area | |  |  |  |  |  |  |  |  |  |  |  |  |
|  | Metropolitan | 11,561 | 50.2% | 1,006 | 50.3% | 4,016 | 50.2% | 3,019 | 50.3% | 1,504 | 50.1% | 2,016 | 50.4% |
|  | Chungcheong | 2,384 | 10.4% | 205 | 10.2% | 828 | 10.3% | 622 | 10.4% | 315 | 10.5% | 414 | 10.3% |
|  | Yeongnam | 2,270 | 9.9% | 196 | 9.8% | 792 | 9.9% | 592 | 9.9% | 297 | 9.9% | 393 | 9.8% |
|  | Honam | 5,807 | 25.2% | 508 | 25.4% | 2,026 | 25.3% | 1,513 | 25.2% | 757 | 25.2% | 1,003 | 25.1% |
|  | None of the above | 996 | 4.3% | 86 | 4.3% | 344 | 4.3% | 261 | 4.3% | 130 | 4.3% | 175 | 4.4% |
| Trust in the current government | |  |  |  |  |  |  |  |  |  |  |  |  |
|  | Approval | 11,368 | 49.4% | 887 | 44.3% | 4,059 | 50.7% | 3,498 | 58.2% | 1,349 | 44.9% | 1,575 | 39.4% |
|  | Disapproval | 9,668 | 42.0% | 967 | 48.3% | 3,372 | 42.1% | 1,923 | 32.0% | 1,368 | 45.6% | 2,038 | 50.9% |
|  | Neither/Nor | 857 | 3.7% | 56 | 2.8% | 261 | 3.3% | 257 | 4.3% | 132 | 4.4% | 151 | 3.8% |
|  | No Opinion | 1,125 | 4.9% | 91 | 4.5% | 314 | 3.9% | 329 | 5.5% | 154 | 5.1% | 237 | 5.9% |
| Political ideology | |  |  |  |  |  |  |  |  |  |  |  |  |
|  | Conservative | 5,822 | 25.3% | 517 | 25.8% | 2,142 | 26.8% | 1,407 | 23.4% | 726 | 24.2% | 1,030 | 25.7% |
|  | Neutral | 6,670 | 29.0% | 576 | 28.8% | 2,261 | 28.2% | 1,745 | 29.0% | 856 | 28.5% | 1,232 | 30.8% |
|  | Liberal | 6,568 | 28.5% | 574 | 28.7% | 2,321 | 29.0% | 1,812 | 30.2% | 829 | 27.6% | 1,032 | 25.8% |
|  | No opinion | 3,958 | 17.2% | 334 | 16.7% | 1,282 | 16.0% | 1,043 | 17.4% | 592 | 19.7% | 707 | 17.7% |
| Affective risk perception | |  |  |  |  |  |  |  |  |  |  |  |  |
|  | Worried | 16,343 | 71.0% | 1,182 | 59.1% | 5,526 | 69.0% | 4,169 | 69.4% | 2,353 | 78.4% | 3,113 | 77.8% |
|  | Not worried | 6,471 | 28.1% | 805 | 40.2% | 2,401 | 30.0% | 1,777 | 29.6% | 634 | 21.1% | 854 | 21.3% |
|  | No opinion | 204 | 0.9% | 14 | 0.7% | 79 | 1.0% | 61 | 1.0% | 16 | 0.5% | 34 | 0.8% |
| Cognitive risk perception | |  |  |  |  |  |  |  |  |  |  |  |  |
|  | Probable | 12,213 | 53.1% | 803 | 40.1% | 3,979 | 49.7% | 3,226 | 53.7% | 1,729 | 57.6% | 2,476 | 61.9% |
|  | Not probable | 9,036 | 39.3% | 1,045 | 52.2% | 3,406 | 42.5% | 2,353 | 39.2% | 1,064 | 35.4% | 1,168 | 29.2% |
|  | No opinion | 1,769 | 7.7% | 153 | 7.6% | 621 | 7.8% | 428 | 7.1% | 210 | 7.0% | 357 | 8.9% |

**S4 Table. General characteristics of respondents by survey**

|  |  | Overall |  | Survey 1 |  | Survey 2 |  | Survey 3 |  | Survey 4 |  | Survey 5 |  |
| --- | --- | --- | --- | --- | --- | --- | --- | --- | --- | --- | --- | --- | --- |
| N | (Respondents) | 23018 |  | 1000 |  | 1001 |  | 1002 |  | 1001 |  | 1000 |  |
| Age (years) | |  |  |  |  |  |  |  |  |  |  |  |  |
|  | Mean | 49.3 |  | 49.5 |  | 49.2 |  | 49.3 |  | 48.7 |  | 49.0 |  |
|  | SD | 16.7 |  | 16.5 |  | 16.4 |  | 16.9 |  | 16.7 |  | 16.9 |  |
|  | 19–29 | 3630 | 15.8% | 149 | 14.9% | 153 | 15.3% | 157 | 15.7% | 156 | 15.6% | 166 | 16.6% |
|  | 30–39 | 3505 | 15.2% | 163 | 16.3% | 161 | 16.1% | 147 | 14.7% | 166 | 16.6% | 156 | 15.6% |
|  | 40–49 | 4397 | 19.1% | 195 | 19.5% | 187 | 18.7% | 195 | 19.5% | 198 | 19.8% | 190 | 19.0% |
|  | 50–59 | 4802 | 20.9% | 210 | 21.0% | 213 | 21.3% | 211 | 21.1% | 200 | 20.0% | 205 | 20.5% |
|  | 60+ | 6684 | 29.0% | 283 | 28.3% | 287 | 28.7% | 292 | 29.1% | 281 | 28.1% | 283 | 28.3% |
| Gender | |  |  |  |  |  |  |  |  |  |  |  |  |
|  | Men | 11613 | 50.5% | 505 | 50.5% | 512 | 51.1% | 520 | 51.9% | 494 | 49.4% | 509 | 50.9% |
|  | Women | 11405 | 49.5% | 495 | 49.5% | 489 | 48.9% | 482 | 48.1% | 507 | 50.6% | 491 | 49.1% |
| Job | |  |  |  |  |  |  |  |  |  |  |  |  |
|  | Unemployed | 2722 | 11.8% | 112 | 11.2% | 107 | 10.7% | 123 | 12.3% | 84 | 8.4% | 119 | 11.9% |
|  | Farming/Forestry/Fishing | 682 | 3.0% | 30 | 3.0% | 29 | 2.9% | 33 | 3.3% | 27 | 2.7% | 28 | 2.8% |
|  | Self-employed | 3339 | 14.5% | 114 | 11.4% | 145 | 14.5% | 133 | 13.3% | 160 | 16.0% | 183 | 18.3% |
|  | Blue-collar | 3552 | 15.4% | 176 | 17.6% | 165 | 16.5% | 153 | 15.3% | 139 | 13.9% | 147 | 14.7% |
|  | White-collar | 7051 | 30.6% | 303 | 30.3% | 323 | 32.3% | 295 | 29.4% | 341 | 34.1% | 290 | 29.0% |
|  | Homemaker and Student | 5579 | 24.2% | 260 | 26.0% | 229 | 22.9% | 258 | 25.7% | 247 | 24.7% | 229 | 22.9% |
| Self-reported household status | |  |  |  |  |  |  |  |  |  |  |  |  |
|  | Upper/Upper Middle | 3617 | 15.7% | 154 | 15.4% | 173 | 17.3% | 132 | 13.2% | 170 | 17.0% | 133 | 13.3% |
|  | Middle | 10331 | 44.9% | 467 | 46.7% | 461 | 46.1% | 497 | 49.6% | 455 | 45.5% | 456 | 45.6% |
|  | Lower Middle/Lower | 9,070 | 39.4% | 379 | 37.9% | 367 | 36.7% | 373 | 37.2% | 376 | 37.6% | 411 | 41.1% |
| Residential area | |  |  |  |  |  |  |  |  |  |  |  |  |
|  | Metropolitan | 11561 | 50.2% | 504 | 50.4% | 502 | 50.1% | 502 | 50.1% | 498 | 49.8% | 503 | 50.3% |
|  | Chungcheong | 2384 | 10.4% | 100 | 10.0% | 105 | 10.5% | 102 | 10.2% | 106 | 10.6% | 102 | 10.2% |
|  | Yeongnam | 2270 | 9.9% | 99 | 9.9% | 97 | 9.7% | 97 | 9.7% | 99 | 9.9% | 98 | 9.8% |
|  | Honam | 5807 | 25.2% | 254 | 25.4% | 254 | 25.4% | 257 | 25.6% | 254 | 25.4% | 254 | 25.4% |
|  | None of the above | 996 | 4.3% | 43 | 4.3% | 43 | 4.3% | 44 | 4.4% | 44 | 4.4% | 43 | 4.3% |
| Trust in the current government | |  |  |  |  |  |  |  |  |  |  |  |  |
|  | Approval | 11368 | 49.4% | 438 | 43.8% | 449 | 44.9% | 446 | 44.5% | 428 | 42.8% | 440 | 44.0% |
|  | Disapproval | 9668 | 42.0% | 482 | 48.2% | 485 | 48.5% | 478 | 47.7% | 511 | 51.0% | 470 | 47.0% |
|  | Neither/Nor | 857 | 3.7% | 33 | 3.3% | 23 | 2.3% | 28 | 2.8% | 28 | 2.8% | 37 | 3.7% |
|  | No Opinion | 1125 | 4.9% | 47 | 4.7% | 44 | 4.4% | 50 | 5.0% | 34 | 3.4% | 53 | 5.3% |
| Political ideology | |  |  |  |  |  |  |  |  |  |  |  |  |
|  | Conservative | 5822 | 25.3% | 249 | 24.9% | 268 | 26.8% | 269 | 26.8% | 239 | 23.9% | 273 | 27.3% |
|  | Neutral | 6670 | 29.0% | 283 | 28.3% | 293 | 29.3% | 266 | 26.5% | 311 | 31.1% | 268 | 26.8% |
|  | Liberal | 6568 | 28.5% | 295 | 29.5% | 279 | 27.9% | 292 | 29.1% | 292 | 29.2% | 274 | 27.4% |
|  | No opinion | 3958 | 17.2% | 173 | 17.3% | 161 | 16.1% | 175 | 17.5% | 159 | 15.9% | 185 | 18.5% |
| Affective risk perception | |  |  |  |  |  |  |  |  |  |  |  |  |
|  | Worried | 16343 | 71.0% | 628 | 62.8% | 554 | 55.3% | 639 | 63.8% | 777 | 77.6% | 737 | 73.7% |
|  | Not worried | 6471 | 28.1% | 365 | 36.5% | 440 | 44.0% | 358 | 35.7% | 213 | 21.3% | 254 | 25.4% |
|  | No opinion | 204 | 0.9% | 7 | 0.7% | 7 | 0.7% | 5 | 0.5% | 11 | 1.1% | 9 | 0.9% |
| Cognitive risk perception | |  |  |  |  |  |  |  |  |  |  |  |  |
|  | Probable | 12213 | 53.1% | 421 | 42.1% | 382 | 38.2% | 416 | 41.5% | 578 | 57.7% | 510 | 51.0% |
|  | Not probable | 9036 | 39.3% | 496 | 49.6% | 549 | 54.8% | 503 | 50.2% | 345 | 34.5% | 415 | 41.5% |
|  | No opinion | 1769 | 7.7% | 83 | 8.3% | 70 | 7.0% | 83 | 8.3% | 78 | 7.8% | 75 | 7.5% |

(continued)

|  |  | Survey 6 |  | Survey 7 |  | Survey 8 |  | Survey 9 |  | Survey 10 | | Survey 11 | |
| --- | --- | --- | --- | --- | --- | --- | --- | --- | --- | --- | --- | --- | --- |
| N | (Respondents) | 1001 |  | 1000 |  | 1001 |  | 1000 |  | 1001 |  | 1004 |  |
| Age (years) | |  |  |  |  |  |  |  |  |  |  |  |  |
|  | Mean | 49.1 |  | 49.1 |  | 49.2 |  | 49.4 |  | 49.6 |  | 49.1 |  |
|  | SD | 16.0 |  | 16.0 |  | 16.1 |  | 16.1 |  | 17.1 |  | 16.8 |  |
|  | 19–29 | 150 | 15.0% | 142 | 14.2% | 140 | 14.0% | 145 | 14.5% | 165 | 16.5% | 159 | 15.8% |
|  | 30–39 | 157 | 15.7% | 163 | 16.3% | 169 | 16.9% | 152 | 15.2% | 141 | 14.1% | 158 | 15.7% |
|  | 40–49 | 201 | 20.1% | 207 | 20.7% | 201 | 20.1% | 205 | 20.5% | 190 | 19.0% | 183 | 18.2% |
|  | 50–59 | 212 | 21.2% | 213 | 21.3% | 208 | 20.8% | 215 | 21.5% | 197 | 19.7% | 210 | 20.9% |
|  | 60+ | 281 | 28.1% | 275 | 27.5% | 283 | 28.3% | 283 | 28.3% | 308 | 30.8% | 294 | 29.3% |
| Gender | |  |  |  |  |  |  |  |  |  |  |  |  |
|  | Men | 498 | 49.8% | 508 | 50.8% | 501 | 50.0% | 505 | 50.5% | 520 | 51.9% | 505 | 50.3% |
|  | Women | 503 | 50.2% | 492 | 49.2% | 500 | 50.0% | 495 | 49.5% | 481 | 48.1% | 499 | 49.7% |
| Job | |  |  |  |  |  |  |  |  |  |  |  |  |
|  | Unemployed | 106 | 10.6% | 116 | 11.6% | 115 | 11.5% | 108 | 10.8% | 112 | 11.2% | 108 | 10.8% |
|  | Farming/Forestry/Fishing | 30 | 3.0% | 22 | 2.2% | 30 | 3.0% | 22 | 2.2% | 29 | 2.9% | 35 | 3.5% |
|  | Self-employed | 145 | 14.5% | 132 | 13.2% | 160 | 16.0% | 157 | 15.7% | 149 | 14.9% | 152 | 15.1% |
|  | Blue-collar | 132 | 13.2% | 157 | 15.7% | 137 | 13.7% | 133 | 13.3% | 160 | 16.0% | 184 | 18.3% |
|  | White-collar | 331 | 33.1% | 336 | 33.6% | 326 | 32.6% | 322 | 32.2% | 301 | 30.1% | 303 | 30.2% |
|  | Homemaker and Student | 253 | 25.3% | 233 | 23.3% | 228 | 22.8% | 254 | 25.4% | 246 | 24.6% | 220 | 21.9% |
| Self-reported household status | |  |  |  |  |  |  |  |  |  |  |  |  |
|  | Upper/Upper Middle | 175 | 17.5% | 158 | 15.8% | 137 | 13.7% | 163 | 16.3% | 143 | 14.3% | 140 | 13.9% |
|  | Middle | 455 | 45.5% | 461 | 46.1% | 446 | 44.6% | 449 | 44.9% | 444 | 44.4% | 454 | 45.2% |
|  | Lower Middle/Lower | 371 | 37.1% | 381 | 38.1% | 418 | 41.8% | 388 | 38.8% | 414 | 41.4% | 410 | 40.8% |
| Residential area | |  |  |  |  |  |  |  |  |  |  |  |  |
|  | Metropolitan | 500 | 50.0% | 501 | 50.1% | 500 | 50.0% | 506 | 50.6% | 506 | 50.5% | 503 | 50.1% |
|  | Chungcheong | 108 | 10.8% | 107 | 10.7% | 105 | 10.5% | 98 | 9.8% | 100 | 10.0% | 103 | 10.3% |
|  | Yeongnam | 99 | 9.9% | 100 | 10.0% | 99 | 9.9% | 99 | 9.9% | 101 | 10.1% | 99 | 9.9% |
|  | Honam | 252 | 25.2% | 251 | 25.1% | 254 | 25.4% | 252 | 25.2% | 252 | 25.2% | 255 | 25.4% |
|  | None of the above | 42 | 4.2% | 41 | 4.1% | 43 | 4.3% | 45 | 4.5% | 42 | 4.2% | 44 | 4.4% |
| Trust in the current government | |  |  |  |  |  |  |  |  |  |  |  |  |
|  | Approval | 496 | 49.6% | 502 | 50.2% | 562 | 56.1% | 569 | 56.9% | 616 | 61.5% | 708 | 70.5% |
|  | Disapproval | 448 | 44.8% | 420 | 42.0% | 382 | 38.2% | 358 | 35.8% | 305 | 30.5% | 211 | 21.0% |
|  | Neither/Nor | 31 | 3.1% | 33 | 3.3% | 29 | 2.9% | 43 | 4.3% | 32 | 3.2% | 33 | 3.3% |
|  | No Opinion | 26 | 2.6% | 45 | 4.5% | 28 | 2.8% | 30 | 3.0% | 48 | 4.8% | 52 | 5.2% |
| Political ideology | |  |  |  |  |  |  |  |  |  |  |  |  |
|  | Conservative | 247 | 24.7% | 304 | 30.4% | 285 | 28.5% | 260 | 26.0% | 265 | 26.5% | 219 | 21.8% |
|  | Neutral | 293 | 29.3% | 268 | 26.8% | 278 | 27.8% | 302 | 30.2% | 275 | 27.5% | 294 | 29.3% |
|  | Liberal | 300 | 30.0% | 267 | 26.7% | 285 | 28.5% | 311 | 31.1% | 300 | 30.0% | 325 | 32.4% |
|  | No opinion | 161 | 16.1% | 161 | 16.1% | 153 | 15.3% | 127 | 12.7% | 161 | 16.1% | 166 | 16.5% |
| Affective risk perception | |  |  |  |  |  |  |  |  |  |  |  |  |
|  | Worried | 694 | 69.3% | 713 | 71.3% | 677 | 67.6% | 684 | 68.4% | 605 | 60.4% | 559 | 55.7% |
|  | Not worried | 297 | 29.7% | 279 | 27.9% | 306 | 30.6% | 306 | 30.6% | 388 | 38.8% | 437 | 43.5% |
|  | No opinion | 10 | 1.0% | 8 | 0.8% | 18 | 1.8% | 10 | 1.0% | 8 | 0.8% | 8 | 0.8% |
| Cognitive risk perception | |  |  |  |  |  |  |  |  |  |  |  |  |
|  | Probable | 538 | 53.7% | 504 | 50.4% | 465 | 46.5% | 490 | 49.0% | 478 | 47.8% | 444 | 44.2% |
|  | Not probable | 386 | 38.6% | 417 | 41.7% | 435 | 43.5% | 453 | 45.3% | 452 | 45.2% | 501 | 49.9% |
|  | No opinion | 77 | 7.7% | 79 | 7.9% | 101 | 10.1% | 57 | 5.7% | 71 | 7.1% | 59 | 5.9% |

(continued)

|  |  | Survey 12 | | Survey 13 | | Survey 14 | | Survey 15 | | Survey 16 | | Survey 17 | |
| --- | --- | --- | --- | --- | --- | --- | --- | --- | --- | --- | --- | --- | --- |
| N | (Respondents) | 1000 |  | 1001 |  | 1001 |  | 1000 |  | 1001 |  | 1002 |  |
| Age (years) | |  |  |  |  |  |  |  |  |  |  |  |  |
|  | Mean | 49.2 |  | 48.5 |  | 49.0 |  | 49.5 |  | 49.3 |  | 49.7 |  |
|  | SD | 17.1 |  | 16.9 |  | 17.2 |  | 17.4 |  | 16.7 |  | 16.6 |  |
|  | 19–29 | 166 | 16.6% | 177 | 17.7% | 168 | 16.8% | 178 | 17.8% | 145 | 14.5% | 151 | 15.1% |
|  | 30–39 | 152 | 15.2% | 144 | 14.4% | 152 | 15.2% | 125 | 12.5% | 173 | 17.3% | 151 | 15.1% |
|  | 40–49 | 182 | 18.2% | 194 | 19.4% | 189 | 18.9% | 186 | 18.6% | 189 | 18.9% | 192 | 19.2% |
|  | 50–59 | 214 | 21.4% | 206 | 20.6% | 203 | 20.3% | 211 | 21.1% | 209 | 20.9% | 210 | 21.0% |
|  | 60+ | 286 | 28.6% | 280 | 28.0% | 289 | 28.9% | 300 | 30.0% | 285 | 28.5% | 298 | 29.7% |
| Gender | |  |  |  |  |  |  |  |  |  |  |  |  |
|  | Men | 499 | 49.9% | 495 | 49.5% | 499 | 49.9% | 514 | 51.4% | 502 | 50.1% | 498 | 49.7% |
|  | Women | 501 | 50.1% | 506 | 50.5% | 502 | 50.1% | 486 | 48.6% | 499 | 49.9% | 504 | 50.3% |
| Job | |  |  |  |  |  |  |  |  |  |  |  |  |
|  | Unemployed | 121 | 12.1% | 112 | 11.2% | 133 | 13.3% | 138 | 13.8% | 123 | 12.3% | 121 | 12.1% |
|  | Farming/Forestry/Fishing | 39 | 3.9% | 21 | 2.1% | 23 | 2.3% | 36 | 3.6% | 30 | 3.0% | 36 | 3.6% |
|  | Self-employed | 136 | 13.6% | 140 | 14.0% | 134 | 13.4% | 138 | 13.8% | 144 | 14.4% | 157 | 15.7% |
|  | Blue-collar | 161 | 16.1% | 152 | 15.2% | 161 | 16.1% | 153 | 15.3% | 155 | 15.5% | 137 | 13.7% |
|  | White-collar | 290 | 29.0% | 318 | 31.8% | 290 | 29.0% | 276 | 27.6% | 305 | 30.5% | 317 | 31.6% |
|  | Homemaker and Student | 249 | 24.9% | 250 | 25.0% | 258 | 25.8% | 252 | 25.2% | 241 | 24.1% | 227 | 22.7% |
| Self-reported household status | |  |  |  |  |  |  |  |  |  |  |  |  |
|  | Upper/Upper Middle | 166 | 16.6% | 168 | 16.8% | 165 | 16.5% | 166 | 16.6% | 161 | 16.1% | 159 | 15.9% |
|  | Middle | 439 | 43.9% | 431 | 43.1% | 444 | 44.4% | 451 | 45.1% | 432 | 43.2% | 454 | 45.3% |
|  | Lower Middle/Lower | 395 | 39.5% | 402 | 40.2% | 392 | 39.2% | 383 | 38.3% | 408 | 40.8% | 389 | 38.8% |
| Residential area | |  |  |  |  |  |  |  |  |  |  |  |  |
|  | Metropolitan | 501 | 50.1% | 502 | 50.1% | 502 | 50.1% | 503 | 50.3% | 508 | 50.7% | 502 | 50.1% |
|  | Chungcheong | 104 | 10.4% | 106 | 10.6% | 104 | 10.4% | 106 | 10.6% | 99 | 9.9% | 104 | 10.4% |
|  | Yeongnam | 99 | 9.9% | 99 | 9.9% | 97 | 9.7% | 99 | 9.9% | 99 | 9.9% | 99 | 9.9% |
|  | Honam | 254 | 25.4% | 250 | 25.0% | 254 | 25.4% | 248 | 24.8% | 252 | 25.2% | 253 | 25.2% |
|  | None of the above | 42 | 4.2% | 44 | 4.4% | 44 | 4.4% | 44 | 4.4% | 43 | 4.3% | 44 | 4.4% |
| Trust in the current government | |  |  |  |  |  |  |  |  |  |  |  |  |
|  | Approval | 655 | 65.5% | 624 | 62.3% | 551 | 55.0% | 498 | 49.8% | 462 | 46.2% | 465 | 46.4% |
|  | Disapproval | 259 | 25.9% | 275 | 27.5% | 355 | 35.5% | 399 | 39.9% | 424 | 42.4% | 458 | 45.7% |
|  | Neither/Nor | 48 | 4.8% | 44 | 4.4% | 39 | 3.9% | 45 | 4.5% | 48 | 4.8% | 39 | 3.9% |
|  | No Opinion | 38 | 3.8% | 58 | 5.8% | 56 | 5.6% | 58 | 5.8% | 67 | 6.7% | 40 | 4.0% |
| Political ideology | |  |  |  |  |  |  |  |  |  |  |  |  |
|  | Conservative | 242 | 24.2% | 209 | 20.9% | 254 | 25.4% | 241 | 24.1% | 242 | 24.2% | 250 | 25.0% |
|  | Neutral | 296 | 29.6% | 319 | 31.9% | 273 | 27.3% | 280 | 28.0% | 283 | 28.3% | 285 | 28.4% |
|  | Liberal | 301 | 30.1% | 305 | 30.5% | 293 | 29.3% | 303 | 30.3% | 285 | 28.5% | 295 | 29.4% |
|  | No opinion | 161 | 16.1% | 168 | 16.8% | 181 | 18.1% | 176 | 17.6% | 191 | 19.1% | 172 | 17.2% |
| Affective risk perception | |  |  |  |  |  |  |  |  |  |  |  |  |
|  | Worried | 665 | 66.5% | 714 | 71.3% | 755 | 75.4% | 740 | 74.0% | 736 | 73.5% | 834 | 83.2% |
|  | Not worried | 320 | 32.0% | 280 | 28.0% | 237 | 23.7% | 249 | 24.9% | 254 | 25.4% | 167 | 16.7% |
|  | No opinion | 15 | 1.5% | 7 | 0.7% | 9 | 0.9% | 11 | 1.1% | 11 | 1.1% | 1 | 0.1% |
| Cognitive risk perception | |  |  |  |  |  |  |  |  |  |  |  |  |
|  | Probable | 528 | 52.8% | 557 | 55.6% | 557 | 55.6% | 578 | 57.8% | 562 | 56.1% | 608 | 60.7% |
|  | Not probable | 405 | 40.5% | 386 | 38.6% | 353 | 35.3% | 350 | 35.0% | 358 | 35.8% | 340 | 33.9% |
|  | No opinion | 67 | 6.7% | 58 | 5.8% | 91 | 9.1% | 72 | 7.2% | 81 | 8.1% | 54 | 5.4% |

(continued)

|  |  | Survey 18 | | Survey 19 | | Survey 20 | | Survey 21 | | Survey 22 | | Survey 23 | |
| --- | --- | --- | --- | --- | --- | --- | --- | --- | --- | --- | --- | --- | --- |
| N | (Respondents) | 1000 |  | 1001 |  | 1001 |  | 1000 |  | 1000 |  | 1000 |  |
| Age (years) | |  |  |  |  |  |  |  |  |  |  |  |  |
|  | Mean | 49.7 |  | 49.1 |  | 50.0 |  | 49.5 |  | 49.9 |  | 49.6 |  |
|  | SD | 16.7 |  | 16.9 |  | 17.0 |  | 17.0 |  | 16.8 |  | 17.2 |  |
|  | 19–29 | 147 | 14.7% | 162 | 16.2% | 157 | 15.7% | 165 | 16.5% | 157 | 15.7% | 175 | 17.5% |
|  | 30–39 | 147 | 14.7% | 159 | 15.9% | 146 | 14.6% | 147 | 14.7% | 140 | 14.0% | 136 | 13.6% |
|  | 40–49 | 194 | 19.4% | 181 | 18.1% | 183 | 18.3% | 189 | 18.9% | 190 | 19.0% | 176 | 17.6% |
|  | 50–59 | 209 | 20.9% | 208 | 20.8% | 211 | 21.1% | 208 | 20.8% | 211 | 21.1% | 208 | 20.8% |
|  | 60+ | 303 | 30.3% | 291 | 29.1% | 304 | 30.4% | 291 | 29.1% | 302 | 30.2% | 305 | 30.5% |
| Gender | |  |  |  |  |  |  |  |  |  |  |  |  |
|  | Men | 501 | 50.1% | 501 | 50.0% | 523 | 52.2% | 498 | 49.8% | 498 | 49.8% | 508 | 50.8% |
|  | Women | 499 | 49.9% | 500 | 50.0% | 478 | 47.8% | 502 | 50.2% | 502 | 50.2% | 492 | 49.2% |
| Job | |  |  |  |  |  |  |  |  |  |  |  |  |
|  | Unemployed | 119 | 11.9% | 135 | 13.5% | 140 | 14.0% | 105 | 10.5% | 125 | 12.5% | 140 | 14.0% |
|  | Farming/Forestry/Fishing | 33 | 3.3% | 28 | 2.8% | 35 | 3.5% | 30 | 3.0% | 29 | 2.9% | 27 | 2.7% |
|  | Self-employed | 123 | 12.3% | 146 | 14.6% | 136 | 13.6% | 172 | 17.2% | 143 | 14.3% | 140 | 14.0% |
|  | Blue-collar | 152 | 15.2% | 166 | 16.6% | 162 | 16.2% | 180 | 18.0% | 140 | 14.0% | 150 | 15.0% |
|  | White-collar | 323 | 32.3% | 303 | 30.3% | 295 | 29.5% | 272 | 27.2% | 302 | 30.2% | 289 | 28.9% |
|  | Homemaker and Student | 248 | 24.8% | 219 | 21.9% | 229 | 22.9% | 238 | 23.8% | 259 | 25.9% | 252 | 25.2% |
| Self-reported household status | |  |  |  |  |  |  |  |  |  |  |  |  |
|  | Upper/Upper Middle | 165 | 16.5% | 143 | 14.3% | 164 | 16.4% | 150 | 15.0% | 160 | 16.0% | 172 | 17.2% |
|  | Middle | 417 | 41.7% | 436 | 43.6% | 448 | 44.8% | 432 | 43.2% | 450 | 45.0% | 452 | 45.2% |
|  | Lower Middle/Lower | 418 | 41.8% | 422 | 42.2% | 389 | 38.9% | 418 | 41.8% | 390 | 39.0% | 376 | 37.6% |
| Residential area | |  |  |  |  |  |  |  |  |  |  |  |  |
|  | Metropolitan | 501 | 50.1% | 501 | 50.0% | 504 | 50.3% | 501 | 50.1% | 504 | 50.4% | 507 | 50.7% |
|  | Chungcheong | 105 | 10.5% | 106 | 10.6% | 103 | 10.3% | 104 | 10.4% | 104 | 10.4% | 103 | 10.3% |
|  | Yeongnam | 99 | 9.9% | 99 | 9.9% | 99 | 9.9% | 99 | 9.9% | 98 | 9.8% | 97 | 9.7% |
|  | Honam | 252 | 25.2% | 252 | 25.2% | 252 | 25.2% | 252 | 25.2% | 250 | 25.0% | 249 | 24.9% |
|  | None of the above | 43 | 4.3% | 43 | 4.3% | 43 | 4.3% | 44 | 4.4% | 44 | 4.4% | 44 | 4.4% |
| Trust in the current government | |  |  |  |  |  |  |  |  |  |  |  |  |
|  | Approval | 450 | 45.0% | 434 | 43.4% | 440 | 44.0% | 377 | 37.7% | 372 | 37.2% | 386 | 38.6% |
|  | Disapproval | 462 | 46.2% | 448 | 44.8% | 456 | 45.6% | 543 | 54.3% | 537 | 53.7% | 502 | 50.2% |
|  | Neither/Nor | 39 | 3.9% | 54 | 5.4% | 43 | 4.3% | 29 | 2.9% | 38 | 3.8% | 41 | 4.1% |
|  | No Opinion | 49 | 4.9% | 65 | 6.5% | 62 | 6.2% | 51 | 5.1% | 53 | 5.3% | 71 | 7.1% |
| Political ideology | |  |  |  |  |  |  |  |  |  |  |  |  |
|  | Conservative | 246 | 24.6% | 230 | 23.0% | 269 | 26.9% | 240 | 24.0% | 266 | 26.6% | 255 | 25.5% |
|  | Neutral | 291 | 29.1% | 280 | 28.0% | 299 | 29.9% | 329 | 32.9% | 303 | 30.3% | 301 | 30.1% |
|  | Liberal | 245 | 24.5% | 289 | 28.9% | 260 | 26.0% | 233 | 23.3% | 264 | 26.4% | 275 | 27.5% |
|  | No opinion | 218 | 21.8% | 202 | 20.2% | 173 | 17.3% | 198 | 19.8% | 167 | 16.7% | 169 | 16.9% |
| Affective risk perception | |  |  |  |  |  |  |  |  |  |  |  |  |
|  | Worried | 797 | 79.7% | 722 | 72.1% | 801 | 80.0% | 804 | 80.4% | 777 | 77.7% | 731 | 73.1% |
|  | Not worried | 196 | 19.6% | 271 | 27.1% | 190 | 19.0% | 187 | 18.7% | 214 | 21.4% | 263 | 26.3% |
|  | No opinion | 7 | 0.7% | 8 | 0.8% | 10 | 1.0% | 9 | 0.9% | 9 | 0.9% | 6 | 0.6% |
| Cognitive risk perception | |  |  |  |  |  |  |  |  |  |  |  |  |
|  | Probable | 560 | 56.0% | 561 | 56.0% | 611 | 61.0% | 627 | 62.7% | 625 | 62.5% | 613 | 61.3% |
|  | Not probable | 358 | 35.8% | 366 | 36.6% | 288 | 28.8% | 276 | 27.6% | 296 | 29.6% | 308 | 30.8% |
|  | No opinion | 82 | 8.2% | 74 | 7.4% | 102 | 10.2% | 97 | 9.7% | 79 | 7.9% | 79 | 7.9% |
