# Supplementary material for: The association between the risk perceptions of COVID-19, trust in the government, political ideologies, and socio-demographic factors: A year-long cross-sectional study in South Korea
Source: PLoS One. 2023 Jun 21;18(6):e0280779. doi: 10.1371/journal.pone.0280779 (PMC10284396; doi:10.1371/journal.pone.0280779)
Supplement: Attachment — Submitted filename: 221214 Response_to_peer_reviewer.docx [file pone.0280779.s003.docx]

**Response to peer reviewer comments**

Dear Natalie J. Shook,

Thank you for giving us the opportunity to submit a 2^nd^ revised draft of our manuscript titled **“The Association among Risk Perceptions of COVID-19, Trust in Government, Political Ideology, and Socio-Demographic Factors: A Year Consecutive Cross-Sectional Study in South Korea” [PONE-D-22-09993R1]** to the *PLOS ONE*. We appreciate the time and effort that you and the reviewers have dedicated to providing valuable feedback on the manuscript. We are also grateful to the reviewers for their insightful comments on the paper. We have been able to incorporate changes in response to a majority of the suggestions provided by the reviewers.

Below is a point-by-point response to the reviewers’ comments and concerns.

**Comments from Reviewer 2**

**Methods**

- 1. Not sure "Factors that were potentially associated with both dimensions of risk perception included sociodemographic factors, trust in the government, and political ideology." this sentence belong under the subtitle demographics. Maybe it should have come right after the Measures title (P8L147).

- Response to the reviewer’s comment:

Thank you for your thorough review. Edited as suggested.

- 2. Since you are asking about the current president and not asking about general attitudes towards government it would be more appropriate to label this variable as "Trust in current government". This point may also be discussed in the discussion section (P8L157).

- Response to the reviewer’s comment:

We are grateful for the precise feedback. We updated P8L157 and the others accordingly.

**Results**

- 3. The analyses plan explains what analyses are conducted. However mentioning the name of the type of tests you conducted before reporting the results would be helpful for readers who are not custom to your analyses. For example you report a p value at p10l203, but it's not clear what test is used. You can also add this info at the tables, I think it would be very helpful for the readers.

- Response to the reviewer’s comment:

Thank you very much for important point. To convey accurate information to readers, we added the type of statistical test we used in each table. In addition, we further specified the type of tests and analysis for Table 1 and Figure 1 in the "Analysis" paragraph in the Method section, like the following:

"... We reported survey response rates over time. The relationships between each factor and risk perception were investigated by univariate analyses with the chi-squared test (categorical variables) and the two-sample t-test (numeric variables). The correlations between the number of confirmed cases and the two dimensions of risk perception were evaluated using Pearson’s correlation coefficient and the t-test for correlation. ...."

- 4. Table 1. The label "Presidential job approval rating" is not consistent with how you labeled this variable in your measures.

- Response to the reviewer’s comment:

We have updated all sentences from "presidential job approval rating" to "trust in the current government."

- 5. Table 3&4. At table 2 you indicated the reference variables, this was not done at Tables 3 and 4. Is there a reason for this difference? If not I think it helps to include that indication.

- Response to the reviewer’s comment:

Thank you for important point. We added indication "1.00 (reference)" into the reference groups in Tables 3&4.

- 6. Overall comment. Labels of gender (men, women) and sex (male, female) are used interchangeably. Please be consistent on the use of this variable.

- Response to the reviewer’s comment:

We are grateful for careful review. The updated version replaced sex (male, female) by the labels of gender (men, women) for consistency.

Lastly, We have added information about the support for YGC’s work (P23l374).
